# PAI-1 regulates AT2-mediated re-alveolarization and ion permeability

**DOI:** 10.1186/s13287-023-03414-4

**Published:** 2023-07-27

**Authors:** Gibran Ali, Mo Zhang, Jianjun Chang, Runzhen Zhao, Yang Jin, Jiwang Zhang, Hong-Long Ji

**Affiliations:** 1 Department of Cellular and Molecular Biology, Texas Lung Injury Institute, University of Texas at Tyler Health Science Center, Tyler, TX USA; 2 Xinxiang Medical University, Xinxiang, Henan China; 3 Division of Pulmonary and Critical Care Medicine, Department of Medicine, Boston University, Boston, MA USA; 4 Department of Cancer Biology, Oncology Institute, Cardinal Bernardin Cancer Center, Loyola University Medical Center, Maywood, IL USA; 5 Department of Surgery, Burn and Shock Trauma Research Institute, Loyola University Chicago, 2160 S 1St Avenue, Maywood, IL 60153 USA

**Keywords:** *Serpine1*, Proliferation, Differentiation, Organoids, Re-alveolarization

## Abstract

**Background:**

Acute lung injury is characterized by overwhelmingly elevated PAI-1 in both lung edema fluid and the circulating system. The role of increased PAI-1, encoded by *Serpine1 gene,* in the regeneration of injured lung epithelium has not been understood completely. This study aimed to investigate the role of *Serpine1* in the regulation of alveolar type 2 epithelial cell (AT2) fate in a humanized mouse line carrying diseased mutants (*Serpine1*^*Tg*^).

**Methods:**

Wild-type (wt) and *Serpine1*^*Tg*^ AT2 cells were either cultured as monolayers or 3D alveolospheres. Colony-forming assay and total surface area of organoids were analyzed. AT1 and AT2 cells in organoids were counted by immunohistochemistry and fluorescence-activated cell sorting (FACS). To test the potential effects of elevated PAI-1 on the permeability in the epithelial monolayers, we digitized the biophysical properties of polarized AT2 monolayers grown at the air–liquid interface.

**Results:**

A significant reduction in total AT2 cells harvested in *Serpine1*^*Tg*^ mice was observed compared with wt controls. AT2 cells harvested from *Serpine1*^*Tg*^ mice reduced significantly over the wt controls. Spheroids formed by *Serpine1*^*Tg*^ AT2 cells were lesser than wt control. Similarly, the corresponding surface area, a readout of re-alveolarization of injured epithelium, was markedly reduced in *Serpine1*^*Tg*^ organoids. FACS analysis revealed a significant suppression in the number of AT2 cells, in particular, the CD44^+^ subpopulation, in *Serpine1*^*Tg*^ organoids. A lesser ratio of AT1:AT2 cells in *Serpine1*^*Tg*^ organoids was observed compared with wt cultures. There was a significant increase in transepithelial resistance but not amiloride inhibition.

**Conclusions:**

Our study suggests elevated PAI-1 in injured lungs downregulates alveolar epithelial regeneration by reducing the AT2 self-renewal, particularly in the CD44^+^ cells.

## Background

*Serpine1* gene is a member of the serine proteinase inhibitor superfamily that encodes plasminogen activator inhibitor-1 (PAI-1), which is an inhibitor of both tissue plasminogen activator (tPA) and urokinase plasminogen activator (uPA). PAI-1 is the principal inhibitor of fibrinolysis, which plays an important role in pulmonary diseases [1, 2]. PAI-1 is synthesized in endothelial cells then stored in platelets and released upon activation [3]. The animal models of bleomycin- or hyperoxia-induced lung injury have shown a pathogenic role for PAI-1 [4]. Patients with acute lung injury (ALI), acute respiratory distress syndrome (ARDS), and COVID-19 have increased PAI-1 in plasma and pulmonary edema fluid, a predictor of early mortality [5, 6]. The plasma PAI-1 in ALI patients was greater than 300 ng/mL [5] and up to 713.3 ng/ml in severe COVID-19 patients [7]. PAI‐1 expression is also elevated significantly in senescent cells [8].

PAI-1 expression increases with aging and aging-related diseases, including idiopathic pulmonary fibrosis [9]. The increased expression of PAI-1 is associated with decreased fibrinolytic activity in bronchoalveolar lavage fluid in ARDS, interstitial lung diseases, and COVID-19 [10]. The elevated PAI-1 level in the patient lungs causes dysregulation of the fibrinolytic system, which is accompanied by an aberrant fibrin deposition [11, 12]. Further, elevated PAI-1 leads to increased apoptosis of alveolar type 1 (AT1) cells but not (myo)fibroblasts, eventually resulting in the pathogenesis of lung fibrosis [13–15]. In the COVID-19 lungs, AT2 cells adopted an inflammation-associated transient progenitor cell state and failed to undergo a transition into AT1 cells, resulting in impaired lung regeneration and dysfunctional AT2 processes (ER stress, telomere instability, progenitor cell arrest, and senescence) [16, 17]. Transforming growth factor-β1-induced excessive PAI-1 mediates the senescence of AT2 cells, as deletion or inhibition of PAI-1 activity blocks this senescent process in bleomycin-exposed mice [18]. In addition to suppression of fibrinolysis, PAI‐1 modulates cell adhesion, relocation, and multiplying in the progress of lung fibrosis [19].

The epithelium lining of regeneratively quiescent lungs is composed of AT2 progenitor and differentiated AT1 cells. Upon AT1 cells were injured, AT2 cells would be activated to proliferate and transdifferentiate into AT1 cells [20]. AT2 cells are more sensitive to injuries than AT1 cells [21]. To replace dysfunctional AT1 cells, AT2 cells may undergo lifelong self-renewal to maintain alveolar epithelial homeostasis [22]. However, the mechanisms for PAI-1 to regulate the regeneration of injured lungs are unclear.

This study aimed at investigating the role of elevated PAI-1 in regulating re-alveolarization by comparing the fate of *Serpine1*^*Tg*^ AT2 cells with wt controls. Here, we have used *Serpine1*^*Tg*^ mice, which is a well-established mouse strain carrying a humanized mutant of *Serpine1* gene expressing 170-fold of PAI-1 over wild type [23]. The extremely elevated PAI-1 level is a biomarker of lung injury. The significantly difference in PAI-1 expression in AT2 cells of wt and *Serpine1*^*Tg*^ mice provides a clinically related model to examine the role of increased PAI-1 in the regulation of AT2 fate. Our results showed that increased PAI-1 affected the proliferative ability of AT2 cells and bioelectric features.

## Methods

### Animal husbandry

Both wt and *Serpine1*^*Tg*^ mice (Jackson Laboratory #007241) were obtained from Jackson Laboratory, USA. *Serpine1*^*Tg*^ mice carrying two human mutants (K154T and Q319L) have more than tripled the functional half-life, and the expression level of PAI-1 is increased approximately 170-fold over wild type [23]. Mice were kept in a pathogen-free facility. Age, sex, and weight-matched (4–12 months) wild-type (wt) and *Serpine1*^*Tg*^ mice were euthanatized for experiments as approved by the Institute of Animal Care and Use Committee of the University of Texas at Tyler Health Science Center.

### Mouse AT2 cell isolation

AT2 cells were isolated from wt and *Serpine1*^*Tg*^ mice as described previously [24]. Briefly, mice were euthanized with overdose ketamine (425 mg/kg) and xylazine (75 mg/kg), intraperitoneally followed by exsanguination and perfusing lungs with DPBS. The trachea was instilled with 50 units/mL dispase followed by 1% low melting point agarose. The lungs were incubated in dispase solution for 45 min at room temperature. The lungs were gently dissociated to the single-cell suspension and passed through a serial filtration (100-, 40-, 30-, and 10-µm cell strainers) and centrifuged at 300 × g for 10 min at 4 °C. Cell suspension was depleted for impurities of CD16/32^+^, CD45^+^, and Ter119^+^ cells. Purified cells were resuspended in a complete mouse medium (CMM: DMEM/F-12 supplemented with 2-mM L-glutamine, 0.25% bovine serum albumin, 10-mM HEPES, 0.1-mM non-essential amino acids, 0.05% ITS, 100-μg/mL primocin, and 10% newborn calf serum). The viability of harvested AT2 cells was assessed by the trypan blue exclusion assay. The purity of isolated AT2 cells was assessed as reported [25].

### *Sorting CD44*^+^*AT2 cells from Serpine1*^*Tg*^*and wt mice*

Freshly isolated cells were seeded on collagen IV-coated plates for 24—36 h to revive CD44 expression diminished by digestive enzymes. Both unattached and attached (trypsinized) cells were collected and blocked with 1% BSA, 4% normal goat serum in PBS. Cells were stained with AF488-EpCAM (BioLegend), APC anti-human CD44 (BioLegend), and their respective isotypes. Cells were sorted using a Beckman Coulter MoFlo high-speed cell sorter. Unstained, isotype, and single-color controls were performed. The gates for CD44 and EpCAM were set based on the results of isotype, and single-color controls were run in parallel. The results were analyzed using FlowJo 10.1.

### Organotypic cultures of AT2 cells

Primary AT2 and MLg2908 cells (ATCC, CCL-206) were co-cultured in Matrigel, as we previously described [24]. The DIC images of organoids (diameter ≥ 50 μm) were visualized with an Olympus IX73 microscope (4X objective, Olympus, Japan). The surface area of individual organoids was measured with ImageJ.

### Quantification of AT1 and AT2 cells in organoids

For analysis of AT2 cell proliferation and differentiation, Matrigel-containing organoids were fixed with 4% paraformaldehyde in PBS for 1 h and immunostained with anti-pdpn and anti-sftpc antibodies to detect AT1 and AT2 cells, respectively. Fluorescent images were projected with a Zeiss LSM 510 confocal microscope and stacked with a Fiji plug-in for ImageJ. Images were stacked for pdpn and sftpc signals separately to count the number of positive cells precisely with a cell counter plug-in of ImageJ. Each slide was scanned for at least six different fields (n = 3 transwells for three independent experiments).

### FACS assays of AT1 and AT2 cells in organoids

AT2 cells are dissociated from organoid cultures from different experimental groups with 10 units/mL dispase and dissociated in 0.25% trypsin–EDTA to get a single-cell suspension. Cells were then stained with antibodies AF488-conjugated EpCAM, APC-conjugated ICAM, and APC-conjugated pdpn. Gates for both colors were set by unstained cells and isotype controls for each antibody. Cells were analyzed by FACSCaliber™ (BD, USA), and the results were analyzed using FlowJow 10.1.

### Culture of polarized AT2 monolayers

Transwell inserts (Costar 3470: 0.4-μm pore size, 0.33 cm^2^ area; Corning Costar, USA) were pre-coated with mouse laminin 1 or 5 at 10 μg/cm^2^ (for mouse AT2 cells; Trevigen, USA) for 4–6 h at 37 °C. Freshly isolated AT2 cells were seeded at 10^6^ cells/cm^2^. The CMM medium (600 μL) was added to the basolateral side of each transwell. The culture medium on the basolateral side was replaced with a serum-free medium 72 h post-seeding. Transepithelial resistance (R_T_, Ω) and potential difference (V_T_, mV) were measured using an epithelial voltohmmeter (EVOM: World Precision Instrument, USA) in 72 h. Cells were cultured for 3 days submerged in culture media and then shifted to the air–liquid interface for another 48–72 h.

### Measurements of bioelectric properties in AT2 monolayers

Transepithelial short-circuit current (Isc, μA/cm^2^) in AT2 monolayers was measured with an 8-channel voltage-clamp amplifier (Physiological Instruments, USA), as we previously described [25]. Briefly, AT2 monolayers were mounted in the vertical Ussing chambers bathed with solutions containing (in mM): 120 NaCl, 25 NaHCO_3_, 3.3 KH_2_PO_4_, 0.83 K_2_HPO_4_, 1.2 CaCl_2_, 1.2 MgCl_2_, 10 HEPES, 10 mannitol (apical compartment), or 10 D-glucose (basolateral compartment). Each solution was iso-osmotic. The transwell cultures bubbled continuously with a gas mixture of 95% O_2_ and 5% CO_2_. The trans-monolayer potential was short-circuited to 0 mV, and a 10-mV pulse of 1-s duration was imposed every 10 s to monitor transepithelial resistance. Data were collected with the acquire and analyze program (version 2.3; Physiologic Instruments). When Isc level reached a plateau, compounds were pipetted to the apical compartment.

### Statistical analysis

Data were presented as mean ± SEM. Normality tests were performed to determine whether the data were parametric or not. If the data were normally distributed and the variance between groups was not significantly different, mean differences in measured variables between the experimental and control groups were assessed with Student’s two-tailed t-tests or one-way ANOVA followed by Tukey’s or Bonferroni’s post hoc test. Otherwise, the Mann–Whitney U-test was applied for analyzing non-parametric results. Two-way ANOVA followed by Sidak’s multiple comparison test was used for multiple comparisons. Mean differences were considered statistically significant at the levels of P < 0.05, P < 0.01, and P < 0.001. Origin Pro 2020 (OriginLab Corporation, USA) was used for the calculation of the power of sample size, statistical analysis, and graphing results.

## Results

### Elevated PAI-1 interrupts homeostasis of alveolar epithelial cells in mouse lungs

**Fig. 1:**
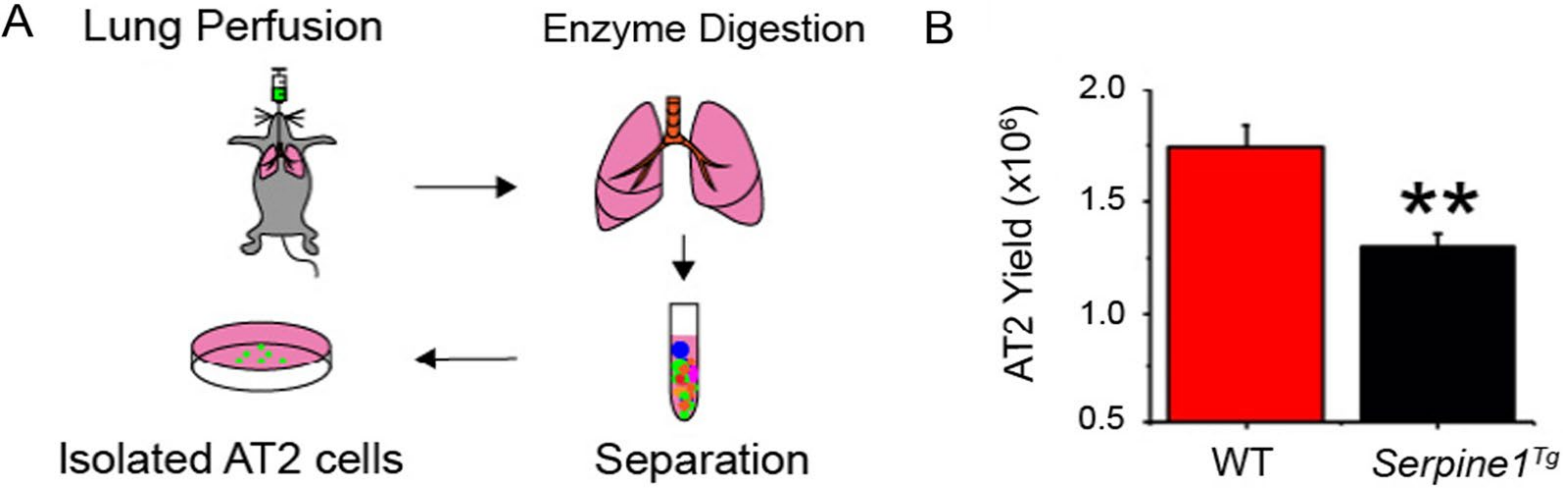
Effects of PAI-1 overexpression on the regeneration of AT2 cells. AT2 cells were isolated from wt and *Serpine1*^*Tg*^ mice overexpressing a gain-of-function of human *Serpine1* gene. **a** Schematic representation of AT2 isolation and (b) average yield of AT2 cells between wt vs. *Serpine1*^*Tg*^ mice (N = 11 pairs of mice). Data are represented as ± SEM, **p < 0.01 vs. wt by two-tailed Student’s t-test

Elevated PAI-1 level is a hallmark of lung injury patients [5, 26–29]. To examine the role of PAI-1 in the regulation of AT2 fate, we compared the yield of AT2 cells between wt and *Serpine1* transgenic (*Serpine1*^*Tg*^) mice. AT2 cells were harvested and purified by perfusing mouse lungs with dispase enzyme and FACS (Fig. 1A). AT2 cells isolated from *Serpine1*^*Tg*^ mice were approximately 1.25 × 10^6^ cells per animal, much lesser compared to that of wt control (1.75 × 10^6^ per mouse, N = 11) (Fig. 1B). These results suggest that the renewal of AT2 cells in mouse lungs could be downregulated by elevated PAI-1.

### Elevated PAI-1 downregulates the fate of AT2 cells

**Fig. 2:**
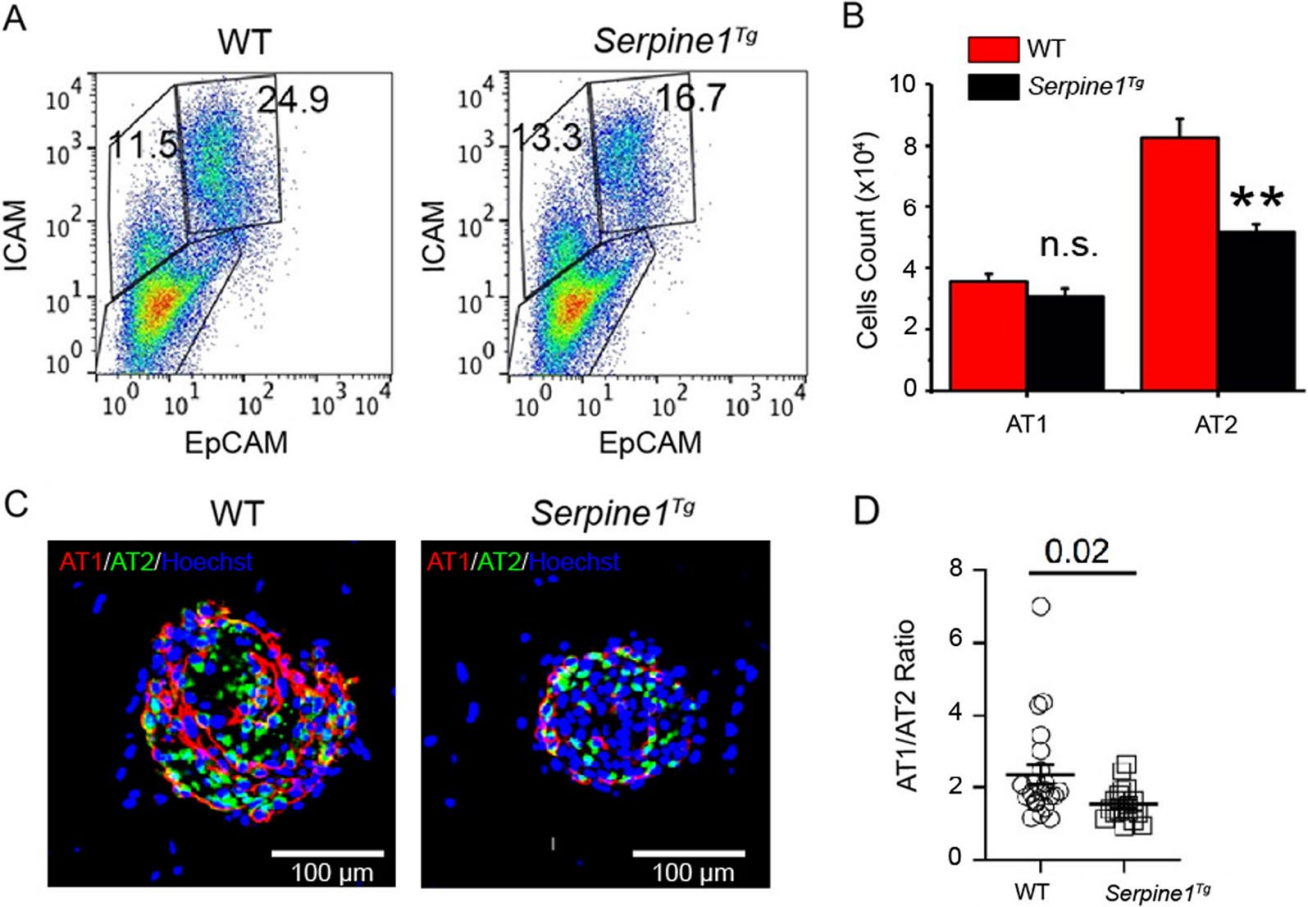
Fibrinolytic niche regulates the fate of AT2 cells in 3D organoids in vitro. **a** FACS analysis of AT1 (ICAM^+^) and AT2 (EpCAM^+^) cells harvested in organoids. **b** Cell count for AT1 and AT2 cells (*N* = 3 pairs of mice). **c** Representative immunofluorescence images of organoids at day 12. Organoids were labeled with pdpn (1:200) and sftpc (1:200) antibodies for AT1 and AT2 cells, respectively. Images of organoids were captured by Zeiss confocal microscope. Images were analyzed using ImageJ software (scale bar 100 µm). **d** Quantitative analysis of AT1–AT2 ratio in organoids between wt and *Serpine1*^*Tg*^ mice (*N* = three technical replicates). Multiple unpaired t-test was used to compare AT1:AT2 ratio between wt and *Serpine1*^*Tg*^ mice. Data expressed as Mean ± SEM. Unpaired t-test and two-tailed *p* < 0.05 were used to compare the means between wt and *Serpine1*^*Tg*^ group

Three-dimensional cultures are a current model to track the lineage of AT2 cells [30, 31]. To analyze the fate of AT2 cells in 3D organoids cultures, we seeded AT2 cells mixed with Mlg fibroblasts in Matrigel for 6–8 days and then quantified AT1 and AT2 cells for differentiation and proliferation, respectively, by immunostaining assays (Fig. 2A and B). The fixed organoids were immunostained with the antibodies for AT1 (pdpn) and AT2 (sftpc) markers (Fig. 2C). AT1 cells formed “luminal” layer, and the outer layer was mainly composed of AT2 cells in medium and large organoids in wt organoids. There was multiple smaller air sac-like hollow organoids observed in *Serpine1*^*Tg*^ cultures compared to the large wt organoids with luminal structures. Moreover, a significantly lesser ratio of AT1 to AT2 cells was observed in *Serpine1*^*Tg*^ organoids compared to wt cultures in a way independent of the size of alveolospheres (Fig. 2D and E). These observations indicate that elevated PAI-1 may downregulate the fate of AT2 cells.

### *Elevated PAI-1 level reduces alveologenesis by targeting CD44*^+^*cells*

**Fig. 3:**
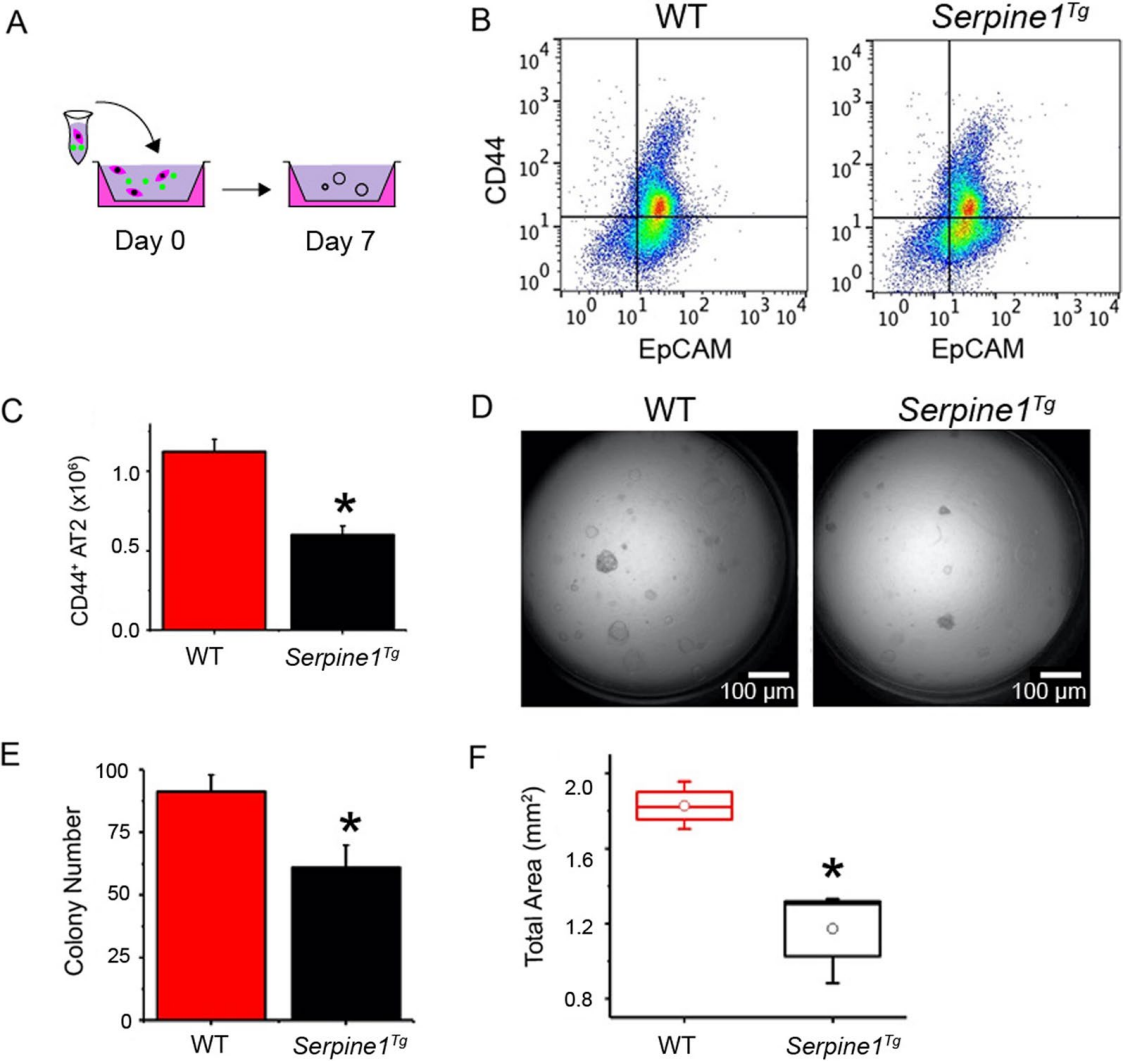
Effect of elevated PAI-1 on re-alveolarization of AT2 cells. **a** Schematic representation of AT2 cells isolation, sorting, and culturing in parallel from wt and *Serpine1*^*Tg*^ mice. **b** FACS comparison of CD44^+^ AT2 cells between wt and *Serpine1*^*Tg*^ mice. **c** Yield of CD44^+^ AT2 cells in vivo between wt and *Serpine1*^*Tg*^ mice (*N* = 9 pairs of mice). **d** DIC images of AT2 organoids for wt and *Serpine1*^*Tg*^ mice. Scale bar, 100 μm. **e** Organoid number and **f** total surface area of organoids per transwell (*n* = 15 transwells per group). Data are represented as ± SEM, *p < 0.05 vs. wt by two-tailed Student’s t-test

We reported that impaired fibrinolytic activity reduced the number of highly proliferative CD44 expressing AT2 cells [24]. The sorted CD44-positive cells were mixed with MLg2908 fibroblasts and cultured for 7 days (Fig. 3A). The harvested AT2 cells were sorted for CD44^+^ and CD44^−^ cells by FACS (Fig. 3B). A significant decrease in CD44^+^ AT2 cells was observed in *Serpine1*^*Tg*^ mice compared to wt mice (Fig. 3C). To analyze the effects of elevated PAI-1 in re-alveolarization in vitro, we grown and quantitated spheroids formed by CD44^+^ AT2 cells (Fig. 3D). Organoids with a diameter equal to or greater than 50 µm were captured as 4 × DIC images 8 days post-seeding. Apparently, the organoids with a diameter ranging from 50 to 200 μm were reduced (N = 12) in *Serpine1*^*Tg*^ cultures compared with wt controls (61 ± 9 organoids vs. 91 ± 7 organoids for wt controls, N = 12, *p* < 0.05) (Fig. 3E). The suppression in organoid formation in *Serpine1*^*Tg*^ cultures was associated with a decrease in the total surface area, a clinical variable for epithelial regeneration (Fig. 3F).

### Elevated PAI-1 affects the bioelectric features in primary AT2 monolayers

**Fig. 4:**
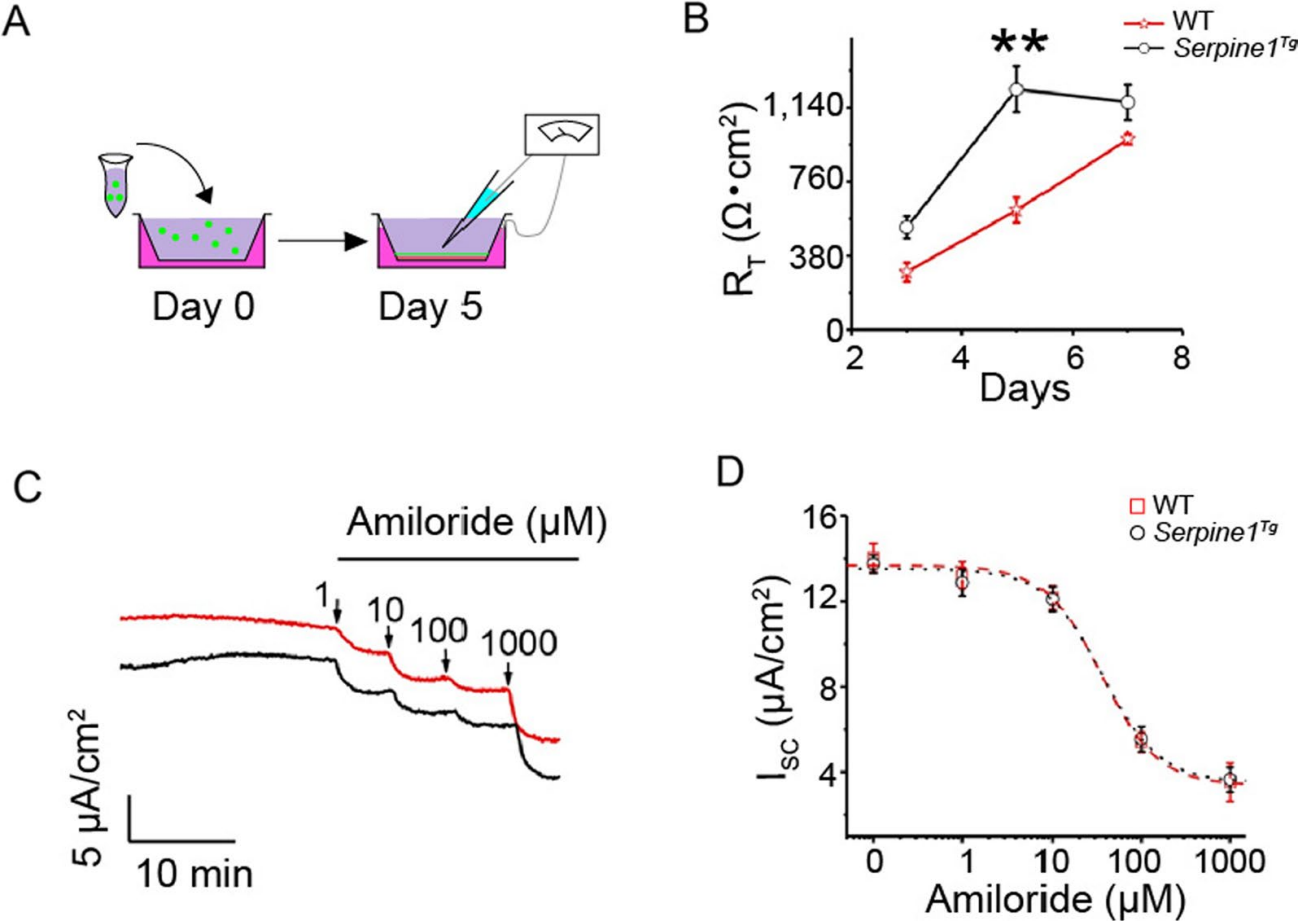
Effect of elevated PAI-1 on the bioelectric features of primary AT2 cells. **a** Schematic representation of AT2 cells monolayer cultures. **b** Transepithelial resistance (R_T_) of AT2 monolayers was measured with a chopstick meter (*N* = 16 monolayers per group). **c** Representatives short-circuit current (Isc) traces in response to accumulating amiloride doses. **d** Raw data from **c** were fitted with the Hill equation to compute the IC_50_ value. *N* = 12. Data are mean ± SEM and analyzed with one-way ANOVA followed by the Tukey post hoc test. *p ≤ 0.05 and **p ≤ 0.01 compared with wt controls

To characterize the effects of elevated PAI-1 in alveolar epithelial cells on the barrier function, we cultured polarized tight AT2 monolayers at the air–liquid interface (Fig. 4A). As we described previously [24], the transepithelial resistance in wt AT2 monolayers was much lesser with a maximal difference on day 5 than that in *Serpine1*^*Tg*^ cultures (Fig. 4B). Basal, amiloride-sensitive, and amiloride-resistant fractions of the short-circuit (I_SC_) currents in *Serpine1*^*Tg*^ monolayers were reduced compared to control (Fig. 4C). However, the amiloride sensitivity remained unchanged between wt and *Serpine1*^*Tg*^ preparations, as shown by apparent *k*_*i*_ values (Fig. 4D).

## Discussion

The primary objective of this study was to decipher the role of elevated PAI-1 in the re-alveolarization mediated by progenitor AT2 cells in injured lungs. We employed humanized *Serpine1*^*Tg*^ mouse strains, 3D organoids, and polarized monolayers of AT2 cells to trace the cell fate. The results demonstrate that elevated PAI-1 results in a significant decline in the proliferative AT2 and differentiated AT1 cells. A significant reduction in AT2 cells in *Serpine1*^*Tg*^ mice and then the formation of organoids was observed as compared to wt cultures. Further, a mild decrease in AT2 to AT1 transition was seen in *Serpine1*^*Tg*^ organoids.

**Fig. 5:**
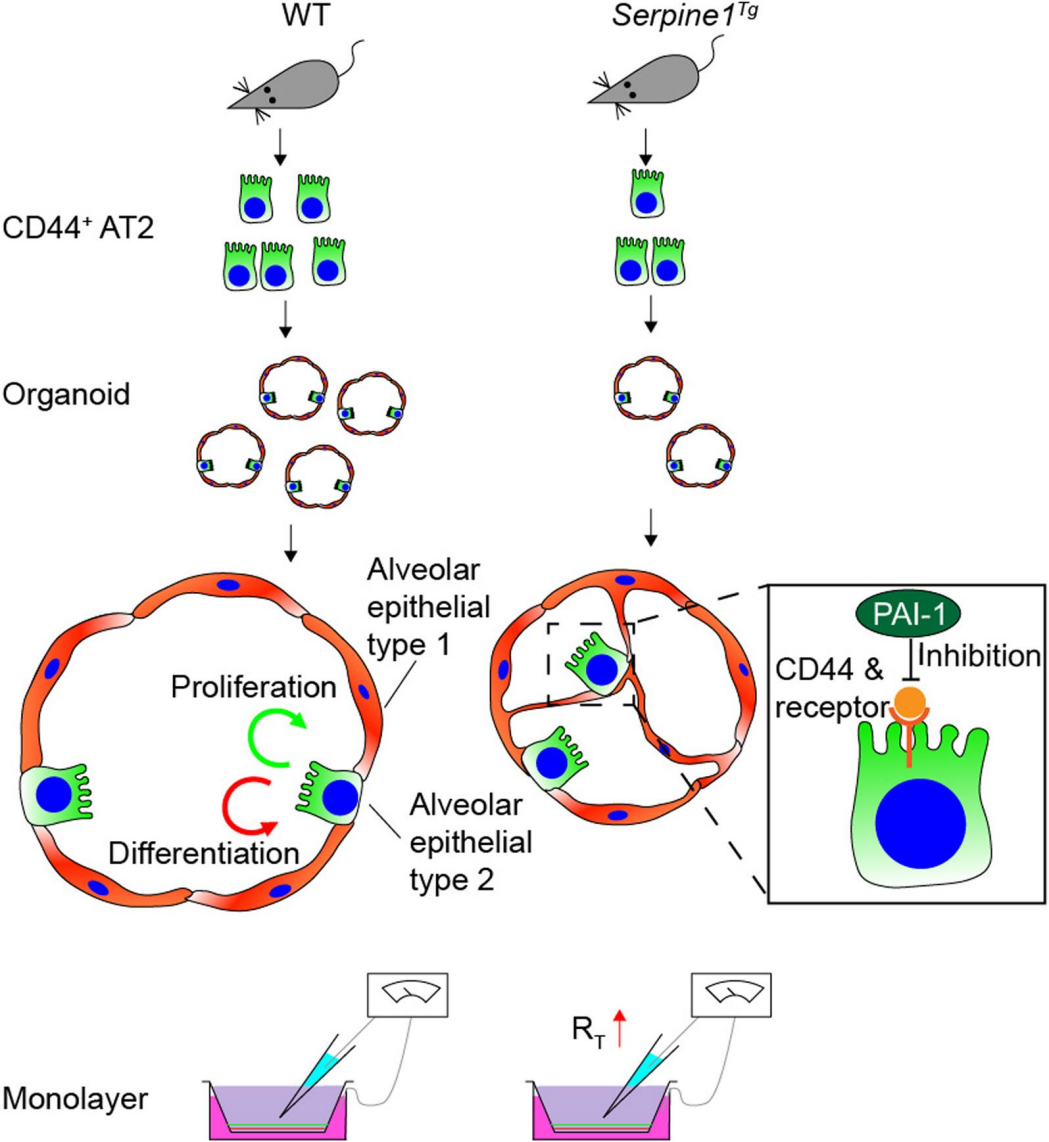
Schematic mechanisms for PAI-1 to regulate the fate of AT2 cells and ion transport

The suppression in AT2 cells could be due to a decrease in highly proliferative CD44^+^ AT2 cells in *Serpine1*^*Tg*^ mice. The supportive evidence comes from organotypic cultures. The sorted CD44^+^ AT2 cells, when mixed with MLg2908 feeder cells, formed a lesser number of spheroids and total surface area in S*erpine1*^Tg^ culture than wt controls. These observations suggest that CD44^+^ AT2 cells could be the downstream target of abnormal PAI-1 in injured lungs (Fig. 5).

PAI-1 overexpression inhibits the proliferation of AT2 cells and differentiation to AT1 cells. This is consistent with our previous studies in *Plau*^*−/−*^ mice [24], and a recent report showed that PAI-1 neutralizing antibody blocked the function of PAI-1, the effects of overexpressed *Serpine1* on the AT2 fate were diminished [32, 33]. On the other hand, *Serpine1* knockout (KO) mice have a lung phenotype of pulmonary clots, which will lead to ischemia, another key regulator of the lineage of stem cells [34]. This confounding factor limits us to use PAI-1 KO mice. Obviously, the fibrinolytic niche balanced by serine proteases and inhibitors finely regulates AT2 cells self-renewal and turnover to AT1 cells. In injured lungs with suppressed plasminogen activators and excessive PAI-1, the lineage of AT2 cells is disrupted, which causes aberrant remodeling of injured lungs. Elevated PAI-1 level is also reported to be associated with epithelial-to-mesenchymal transition (EMT) of AT2 cells in inured lungs and in vitro. EMT is an important mechanism in metastasis and pulmonary fibrosis during the repair of injured lungs [35, 36]. Thus, the reduced AT2 yield in *Serpine1*^*Tg*^ mice could be partially due to EMT. Further, Wnt5a/β catenin along with Notch and Hedgehog signaling pathway is crucial in regulating the AT1–AT2 differentiation. Jain et al. have described that the elevated PAI-1 regulates AT2 proliferation and differentiation via this Wnt5a/β catenin cascade [32].

CD44 receptors could be a key player for elevated PAI-1 to alter the lineage of AT2 cells. uPA-A6-CD44^+^-ENaC cascade regulates the fate of AT2 cells in re-alveolarization [24]. uPA possesses a A6 motif showing high affinity with CD44 receptors [37, 38]. Elevated PAI-1 may interrupt the uPA-CD44 receptor binding competitively. Consequent re-alveolarization could be the critical mechanism for the more susceptible to lung fibrosis post-bleomycin injury in *Plau*^*−/−*^ and *Serpine1*^*Tg*^ mice [39, 40]. Clinically, elevated PAI-1 is a prognostic marker for the outcomes of ARDS patients [41, 42]. In addition to the regulation of uPA-A6-CD44^+^-ENaC cascade, increased PAI-1 level may affect the AT2 fate through β-catenin in a fibroblast-independent manner [32].

*Serpine1* increased transepithelial permeability. The proteolytic link between the fibrinolytic niche and ENaC proteins has been provided by other groups and us [43–46]. uPA cleaves human γENaC subunits to alter ion transport across the airway epithelium [43]. Our new evidence in AT2 monolayers substantiates the regulation of bioelectrical features by PAI-1. The slight diversity between the *Plau*^*−/−*^ and *Serpine1*^*Tg*^ models could be due to their differential regulation of ion transport and stem cell fate.

There are certain limitations of the *Serpine1*^*Tg*^ mouse model. Alternatively, overexpression of *Serpine1* in wt AT2 cells could be applied to validate the observations in the *Serpine1*^*Tg*^ preparations. However, the feasibility of overexpressing *Serpine1* gene in primary AT2 cells is most unlikely doable. Of note, the transfection efficiency is uncertain in primary AT2 cells grown in 3D gel. It is most likely that there would be a technical obstacle for the transfection vector to penetrate Matrigel to reach residential AT2 cells in a gradient or distance-independent manner (47). Uneven distribution of transfection vector might result in “pseudo” phenotypes of AT2 cells.

## Conclusions

In summary, our study uncovers a novel role of elevated PAI-1 in the regeneration of injured lungs. PAI-1 could be a promising pharmaceutic target to accelerate the reparative processes of injured alveolar epithelium in ARDS.

## Data Availability

All data generated or analyzed during this study are included in this published article.

## Abbreviations

3D: Three-dimension
ALI: Acute lung injury
APC: Allophycocyanin
ARDS: Acute respiratory distress syndrome
AT1: Alveolar type 1 epithelial cells
AT2: Alveolar type 2 epithelial cells
BSA: Bovine serum albumin
COVID-19: Coronavirus disease 2019
CMM: Complete mouse medium
DIC: Differential interference contrast
DMEM: Dulbecco's modified eagle medium
DPBS: Dulbecco's phosphate-buffered saline
ENaC: Epithelial sodium channels
EpCAM: Epithelial cellular adhesion molecule
ER: Endoplasmic reticulum
EVOM: Epithelial voltohmmeter
FACS: Fluorescence-activated cell sorting
HEPES: N-2-hydroxyethylpiperazine-N-2-ethane sulfonic acid
ICAM: Intercellular adhesion molecule
Isc: Short-circuit current
ITS: Insulin–transferrin–selenium
PAI-1: Plasminogen activator inhibitor 1
PBS: Phosphate-buffered saline
SEM: Standard error of mean
Tg: Transgenic
tPA: Tissue-type plasminogen activator
uPA: Urokinase plasminogen activator
WT: Wild type
